# Quantum Phonon Transport in Nanomaterials: Combining Atomistic with Non-Equilibrium Green’s Function Techniques

**DOI:** 10.3390/e21080735

**Published:** 2019-07-27

**Authors:** Leonardo Medrano Sandonas, Rafael Gutierrez, Alessandro Pecchia, Alexander Croy, Gianaurelio Cuniberti

**Affiliations:** 1 Institute for Materials Science and Max Bergmann Center of Biomaterials, TU Dresden, 01062 Dresden, Germany; 2 Center for Advancing Electronics Dresden, TU Dresden, 01062 Dresden, Germany; 3 Consiglio Nazionale delle Ricerche, ISMN, Via Salaria km 29.6, Monterotondo, 00017 Rome, Italy; 4 Dresden Center for Computational Materials Science (DCMS), TU Dresden, 01062 Dresden, Germany

**Keywords:** phonon transport, nanostructured materials, green’s functions, density-functional tight binding, Landauer approach, time-dependent transport

## Abstract

A crucial goal for increasing thermal energy harvesting will be to progress towards atomistic design strategies for smart nanodevices and nanomaterials. This requires the combination of computationally efficient atomistic methodologies with quantum transport based approaches. Here, we review our recent work on this problem, by presenting selected applications of the PHONON tool to the description of phonon transport in nanostructured materials. The PHONON tool is a module developed as part of the Density-Functional Tight-Binding (DFTB) software platform. We discuss the anisotropic phonon band structure of selected puckered two-dimensional materials, helical and horizontal doping effects in the phonon thermal conductivity of boron nitride-carbon heteronanotubes, phonon filtering in molecular junctions, and a novel computational methodology to investigate time-dependent phonon transport at the atomistic level. These examples illustrate the versatility of our implementation of phonon transport in combination with density functional-based methods to address specific nanoscale functionalities, thus potentially allowing for designing novel thermal devices.

## 1. Introduction

The accelerated pace of technological advances, which has taken place over the last half-century, has driven the continuous search for higher speed and cheaper computing with the concomitant developments of larger integration densities and miniaturization trends based on novel materials and processes [1]. The negative counterpart of this amazing technological developments consists in the increasing problems with the thermal management, which is ultimately leading to limiting the efficiency of many of these technological advances, mostly in the domain of nanoelectronics. As a response to this challenge, a novel field, nanophononics, has emerged, providing a large variety of interesting physical effects and potential applications [2,3,4,5]. The major goal of nanophononics is to develop efficient strategies for controlling the *heat flux* in organic and inorganic nanostructured materials, and it was originally aiming at realizing thermal devices such as diodes, logic gates, and thermal transistors [2,6]. However, more recent efforts in the field have triggered radically new applications in nanoelectronics [7,8], renewable energy harvesting [9,10], nano- and optomechanical devices [11], quantum technologies [12,13], and therapies, diagnostics, and medical imaging [14].

An important milestone in the field was the theoretically predicted [15] and subsequently measured *quantization* of the phononic thermal conductance in mesoscopic structures [16] at low temperatures, this result building the counterpart of the well-known quantization of the electrical conductance in quantum point contacts and other nanostructures (with conductance quantum given by $e^{2}/h$, *e* being the electron charge and *h* Planck constant). Thermal conductance quantization has also been found in smaller nanostructures, such as gold wires, where quantized thermal conductance at room temperature was shown down to single-atom junctions [17,18]. The issue is, however, still a subject of debate (see, e.g., [19] for a recent discussion). In contrast to the electrical conductance quantization, the quantum of thermal conductance $\kappa_{0}$ depends, however, on the absolute temperature *T* through: $\kappa_{0}=\pi^{2}k_{B}^{2}\;T/3h$, with $k_{B}$ being the Boltzmann constant. This highlights a first important difference between charge and phonon transport. The second one is related to the different energy windows determining the corresponding transport properties: for electrons, the important window lies around the Fermi level, while, for phonons, the conductance results from an integral involving the full vibrational spectrum. Working with a broad spectrum of excitations poses major challenges when it comes to designing thermal devices such as cloaks and rectifiers [2,4], or for information processing in phonon-based computing [6].

From the experimental perspective, it is obviously more difficult to tune heat flow than electrical currents. Unlike electrons, phonons are quasi-particles with zero mass and zero charge, thus they cannot be directly controlled through electromagnetic fields in a straightforward way. Moreover, while considerable progress has been achieved in nanoelectronics in the implementation of local electrodes and gates over very short length scales, establishing temperature gradients over nanoscopic length scales remains a considerable challenge. In this respect, for characterizing thermal devices, novel sophisticated experimental techniques have been developed, such as the 3ω method [20] and the frequency domain thermoeflectance [21], pioneered by Cahill et al. [22]. This has led, in turn, to the modification of atomic force microscopes for thermometry [17,23] and to the development of Scanning Thermal Microscopy [18,24].

Turning nanophononics into a practical field with specific applications in energy management requires nanoscale engineering of the thermal transport properties. This approach has been successfully implemented in nanostructured thermoelectric materials. Even though the fundamental tool to understand nanophononics—non-equilibrium thermodynamics—is well established at the macroscale, many open issues remain at the nanoscale, having deep consequences for the development of relevant strategies to control heat transport in low dimensions. Low-dimensional materials have finite cross sections along one or more spatial dimensions and a large surface-to-volume ratio; as a result, their vibrational spectrum and, consequently, the heat transport mechanisms can be dramatically modified (see the recent reviews in [2,3,4]). Different simulation tools have been used to study heat transport in nanomaterials [4,25]. These approaches can be grouped into three categories, although overlaps between them are clearly possible. The first group includes methodologies based on molecular dynamics (MD) simulations, the equilibrium versions (EMD) based on the Green–Kubo formula [26], and the non-equilibrium versions (NEMD) exploiting Fourier’s law [27,28]. The second category includes approaches based on the Boltzmann transport equation (BTE) [29,30] and lattice dynamics (LD) [31]. Finally, the last category covers methodologies relying on the Landauer approach, or more generally on non-equilibrium Green’s functions (NEGF) [32,33,34,35]. All these methodologies have found extensive application in the prediction of the thermal transport properties of various low-dimensional materials, yielding correct trends and results in good agreement with experimental studies [36,37,38]. As it turns out, the thermal response is sensitively determined by different parameters such as surface boundaries, overall device geometry, spatial confinement, doping, and structural defects [37,39,40,41,42,43].

Moreover, in non-stationary situations with time-dependent external parameters able to affect the transport characteristics, phonon dynamics becomes crucial. For instance, time-varying temperature fields [44,45] or local heating mediated by laser fields [46,47] can be exploited to exert additional control over thermal transport. Thus, novel non-equilibrium effects such as heat pumping [48,49], cooling [50], and rectification [51,52] have been theoretically proposed. The description of such phenomena requires in many instances to work in the time domain, which is very challenging from a numerical point of view. Although noticeable progress has been achieved in dealing with time-dependent spin [53,54] and electron [55,56,57,58,59,60] transport, much less attention has been paid to vibrational degrees of freedom [61,62,63].

Despite the previously delineated methodological advances to model and understand nanoscale thermal transport, there are many basic questions about thermal management of thermoelectric materials, phononic devices, and integrated circuits that must be addressed. In the current paper, we review our recently implemented atomistic models based on the NEGF technique, allowing to address transient and steady quantum phonon transport in low-dimensional systems. We have successfully used our methodology to propose different routes for improved thermal management, eventually leading to realizing novel nanoscale applications.

The paper is organized as follows. In Section 2, the basics of the NEGF approach to compute quantum ballistic transport are introduced. We proceed then to review few selected applications by using the NEGF in combination with a Density-Functional based Tight-Binding approach (DFTB), which allows addressing nanostructures at the atomistic level with considerable accuracy and large computational efficiency. The reviewed applications include 2D materials, BNC heteronanotubes, and molecular junctions. In Section 3, the NEGF formalism previously introduced is expanded to deal with time-dependent thermal transport by exploiting an auxiliary mode approach. This methodology is illustrated for a one-dimensional chain and simple nanoscale junctions based on polyethylene and polyacetylene dimers.

## 2. DFTB-Based Quantum Transport

### 2.1. Ballistic Phonon Transport

One of the most powerful methodologies to study quantum (thermal) transport is the non-equilibrium Green’s functions (NEGF) formalism [35,64,65]. The NEGF method has its origin in quantum field theory [66], and has been developed to study many-particle quantum systems under both equilibrium and nonequilibrium conditions. Different formulations were derived during the early 1960s [67,68,69]. Thus, Keldysh developed a diagrammatic approach, Kadanoff and Baym formulated their approach based on equations of motion. Both methods are suitable for studying a dynamic system in nonequilibrium. For instance, by using the Keldysh formalism, one can obtain formal expressions for the current and electron density [70]. The method has also been successfully used to study electron transport properties in open quantum systems [71,72]. Moreover, NEGF has been recently used on thermal transport investigations not only in the ballistic regime [73,74,75], but also including phonon–phonon scattering [76,77,78,79]. In this section, we describe the NEGF formalism to address ballistic phonon transport in nanostructures. Phonon–phonon interactions require the inclusion of extra self-energy terms, which depend on products of single-phonon Green’s functions, so that the whole problem must be solved self-consistently; however, this goes beyond the scope of this review (see [35,64] for more details concerning this point).

**Figure 1:**
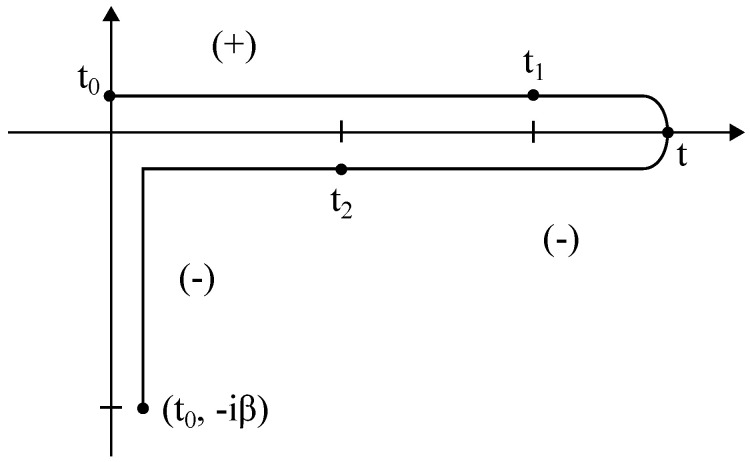
Schwinger/Keldysh contour *C* in the imaginary time plane, $C=\{t\in C,ℜ\;t\in \left[t_{0},\infty \right]ℑ\;t\in \left[t_{0},-\beta \right]\}$. For more clarity, the different contour branches are displayed slightly off the axes. Time-ordering: time $t_{2}$ is later on the contour than time *t*, and *t* is larger than $t_{1}$ [80].

The main difference between the NEGF formalism and ordinary equilibrium theory is that all time-dependent functions are defined on the so-called Schwinger-Keldysh contour (see Figure 1). However, a simplification occurs when $t_{0}\to -\infty$ (Keldysh contour). If the interactions are switch on adiabatically, the contribution from the $\left[t_{0},t_{0}+\beta \right]$ piece vanishes. The information lost by this procedure is related to initial correlations. In many physical situations, such as in steady state transport, it is a plausible approximation to assume that initial correlations have been washed out by the interactions when one reaches the steady state. On the contrary, for the transient response, the role of initial correlations may be important (see Section 3). Here, we consider the Keldysh contour consisting of two branches running from −∞ to *∞* and from *∞* to −∞. Therefore, one can introduce a contour-ordered Green’s function as [80]:

(1) $$G\left(\tau ,\tau^{\prime }\right)=-i\langle T_{C}A\left(\tau \right)B^{T}\left(\tau^{\prime }\right)\rangle ,$$

with $T_{C}$ as the contour-order operator. Based on it, six real-time Green’s functions can be defined [35]:

- The lesser GF, $G^{<}\left(t,t^{\prime }\right)=-i\langle A\left(t^{\prime }\right)B^{T}\left(t\right)\rangle$.
- The greater GF, $G^{>}\left(t,t^{\prime }\right)=-i\langle A\left(t\right)B^{T}\left(t^{\prime }\right)\rangle$.
- The retarded GF, $G^{r}\left(t,t^{\prime }\right)=-i\Theta \left(t-t^{\prime }\right)\langle [A\left(t\right),B^{T}\left(t^{\prime }\right)]\rangle$.
- The advanced GF, $G^{a}\left(t,t^{\prime }\right)=i\Theta \left(t^{\prime }-t\right)\langle [A\left(t\right),B^{T}\left(t^{\prime }\right)]\rangle$.
- The time-ordered GF, $G^{t}\left(t,t^{\prime }\right)=\Theta \left(t-t^{\prime }\right)G^{>}\left(t,t^{\prime }\right)+\Theta \left(t^{\prime }-t\right)G^{<}\left(t,t^{\prime }\right)$.
- The anti-time-ordered GF, $G^{\bar{t}}\left(t,t^{\prime }\right)=\Theta \left(t^{\prime }-t\right)G^{>}\left(t,t^{\prime }\right)+\Theta \left(t-t^{\prime }\right)G^{<}\left(t,t^{\prime }\right)$.

A(t) and B(t) are operators in the Heisenberg picture and Θ(t) is the Heaviside step function. The angular brackets denote trace with the canonical density matrix, i.e., $\langle \cdots \rangle =\mathrm{Tr}\left(\rho \cdots \right)$, with $\rho =e^{-\beta H}/\mathrm{Tr}\left(e^{-\beta H}\right)$ and $\beta =1/(k_{B}T)$, and *H* is the Hamiltonian of the system. The notation $\langle [A,B^{T}]\rangle$ represents a matrix and should be understood as $\langle AB^{T}\rangle -{\langle BA^{T}\rangle }^{T}$.

In equilibrium or non-equilibrium steady state, the Green’s functions only depend on the time difference, $t-t^{\prime }$. The Fourier transform of $G^{r}\left(t-t^{\prime }\right)=G^{r}\left(t,t^{\prime }\right)$ is defined as $G^{r}\left[\omega \right]=\int_{-\infty }^{+\infty }G^{r}\left(t\right)e^{i\omega t}dt$. Using the basic definitions, the following linear relations hold in both frequency and time domains [35]:

(2) $$\begin{array}{cc} G^{r}-G^{a}= & G^{>}+G^{<},\\ G^{t}+G^{\bar{t}}= & G^{>}+G^{<},\\ G^{t}-G^{\bar{t}}= & G^{r}+G^{a}. \end{array}$$

Only three of the six Green’s functions are linearly independent. In systems with time translational invariance, the functions $G^{r}$ and $G^{a}$ are related by $G^{a}\left[\omega \right]={\left(G^{r}\left[\omega \right]\right)}^{†}$. Hence, under general non-equilibrium steady-state conditions, only two are independent, with a typical choice of working with $G^{r}$ and $G^{<}$, although other combinations are possible. Extra relations are defined in the frequency domain for bosons [81]:

(3) $$\begin{array}{cc} G^{<}{\left[\omega \right]}^{†}= & -G^{<}\left[\omega \right],\\ G^{r}\left[-\omega \right]= & G^{r}{\left[\omega \right]}^{*},\\ G^{<}\left[-\omega \right]= & G^{>}{\left[\omega \right]}^{T}=-G^{[}{\omega ]}^{*}+G^{r}{\left[\omega \right]}^{T}-G^{r}{\left[\omega \right]}^{*}. \end{array}$$

Therefore, based on the last two equations, only the positive frequency part of the functions is needed. Equations (2) and (3) are generally valid for non-equilibrium steady states. However, for systems in thermal equilibrium satisfying the fluctuation–dissipation theorem [82], there is an additional equation relating $G^{r}$ and $G^{<}$:

(4) $$G^{<}\left[\omega \right]=f\left(\omega \right)\left(G^{r}\left[\omega \right]-G^{a}\left[\omega \right]\right),$$

where $f\left(\omega \right)={\left(e^{\frac{\hbar \omega }{k_{B}T}}-1\right)}^{-1}$ is the Bose–Einstein distribution function at temperature *T*. $k_{B}$ is the Boltzmann constant. Indeed, the correlation function $G^{<}$ contains information of fluctuations, while $G^{r}-G^{a}$ describes dissipation of the system. $G^{>}\left[\omega \right]=e^{\beta \omega }G^{<}\left[\omega \right]$ also applies for equilibrium systems and, consequently, there is only one independent Green’s function under equilibrium conditions.

**Figure 2:**
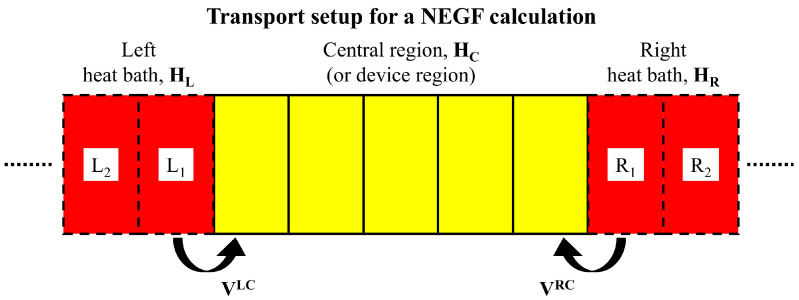
Schematic representation of the common partitioning scheme for phonon transport calculation using Green’s function technique. The entire system is split into three regions: central region and, left and right heat baths. Each of this region are characterized by their own Hamiltonian $H_{\alpha }$ with α=L,C,R. The coupling matrices between heat baths and central region are $V^{LC}$ and $V^{RC}$.

In phonon transport calculations, a partitioning scheme is applied, consisting in splitting the whole system in three regions: one central region (also denoted as device region), connected to two thermal baths on the left (L) and right (R) (see Figure 2). At the simplest level, the thermal baths can be considered as a collection of non-interacting harmonic oscillators. All elastic and/or inelastic scattering processes are therefore assumed to be confined to the central (or device) region. Since we focus on thermal transport mediated by the vibrational system, the phonon Hamiltonian of the whole system is given by [80]:

(5) $$H=\sum_{\alpha =L,C,R}H_{\alpha }+{\left(u^{L}\right)}^{T}V^{LC}u^{C}+{\left(u^{C}\right)}^{T}V^{CR}u^{R}+V_{n},$$

where $H_{\alpha }=\frac{1}{2}{\left({\dot{u}}^{\alpha }\right)}^{T}{\dot{u}}^{\alpha }+\frac{1}{2}{\left(u^{\alpha }\right)}^{T}K^{\alpha }u^{\alpha }$ represents the Hamiltonian of the region α; α=L,C,R, for the left, center, and right regions, respectively. $u^{\alpha }$ is a column vector consisting of all the displacement variables in region α, and ${\dot{u}}^{\alpha }$ is the corresponding conjugate momentum. The following transformation of coordinates has been considered, $u_{j}=\sqrt{m_{j}}x_{j}$, where $x_{j}$ is the relative displacement of *j*th degree of freedom. $K^{\alpha }$ is the mass-reduced force constant matrix. This matrix is the mass-weighted second derivative of energy with respect to displacement at the equilibrium positions:

(6) $${\left[K_{\gamma \beta }\right]}_{ij}=K_{ij}^{\gamma \beta }=\frac{1}{\sqrt{m_{i}m_{j}}}\frac{\partial^{2}E}{\partial u_{i}^{\gamma }u_{j}^{\beta }}.$$

$V^{LC}={\left(V^{CL}\right)}^{T}$ is the coupling matrix between the left lead to the central region; and similarly for $V^{CR}$. The last term $V_{n}$ represents possible many-body interactions, such as phonon–phonon interaction [35]. It contains higher order (higher than 2) derivatives of the energy with respect to the displacements, evaluated at the equilibrium positions.

The most important quantity to calculate is the heat flux *J*, which is defined as the energy transferred from the heat source to the junction in a unit time, and is equal to the energy transferred from the junction to the heat sink in a unit time. Here, it is assumed that no energy is accumulated in the junction. According to this definition, the heat flux out of the left lead is:

(7) $$J_{L}=-\langle {\dot{H}}_{L}\left(t\right)\rangle =i\langle \left[H_{L}\left(t\right),H\right]\rangle =i\langle \left[H_{L}\left(t\right),V^{LC}\left(t\right)\right]\rangle .$$

In the steady state, energy conservation means that $J_{L}+J_{R}=0$. For simplicity, we set ℏ=1. Using Heisenberg’s equation of motion, $J_{L}$ can be written as:

(8) $$\begin{array}{c} \begin{array}{cc} J_{L}= & \langle {\left({\dot{u}}^{L}\right)}^{T}\left(t\right)V^{LC}u^{C}\left(t\right)\rangle \\ = & \lim_{t^{\prime }\to t}\sum_{j,k}V_{jk}^{LC}\langle {\left({\dot{u}}_{j}^{L}\right)}^{T}\left(t^{\prime }\right)u_{k}^{C}\left(t\right)\rangle . \end{array} \end{array}$$

Thus, the heat flux depends on the expectation value of ${\left({\dot{u}}_{j}^{L}\right)}^{T}\left(t^{\prime }\right)u_{k}^{C}\left(t\right)$, which can be written in terms of the lesser Green’s function $G_{CL}^{<}\left(t,t^{\prime }\right)=-i{\langle u^{L}\left(t^{\prime }\right)u^{C}{\left(t\right)}^{T}\rangle }^{T}$. Since operators *u* and $\dot{u}$ are related in Fourier space (frequency domain) as $\dot{u}\left[\omega \right]=-i\omega u\left[\omega \right]$, the derivative is eliminated and one obtains:

(9) $$J_{L}=-\frac{1}{2\pi }\int_{-\infty }^{\infty }\mathrm{Tr}\left(V^{LC}G_{CL}^{<}\left[\omega \right]\right)\omega d\omega .$$

The Green’s functions of interacting systems can be efficiently obtained by solving their equations of motion (EOM) [64]. In this section, EOMs will only be used to obtain expressions for retarded and lesser GFs of the central region. This topic will be expanded with more details in Section 3. First, we have that the contour-ordered GF $G\left(\tau ,\tau^{\prime }\right)=-i\langle T_{\tau }u\left(\tau \right)u{\left(\tau^{\prime }\right)}^{T}\rangle$ satisfies the following equation:

(10) $$-\frac{\partial^{2}G\left(\tau ,\tau^{\prime }\right)}{\partial \tau^{2}}-KG\left(\tau ,\tau^{\prime }\right)=I\delta \left(\tau ,\tau^{\prime }\right)$$

The equation per each region is obtained by partitioning the matrices *G* and *K* into submatrices $G^{\alpha ,\alpha^{\prime }}$ and $K^{\alpha ,\alpha^{\prime }}$, $\alpha ,\alpha^{\prime }=L,C,R$. The free Green’s function for the decoupled system *g* is easily obtained by solving:

(11) $$-\frac{\partial^{2}g^{\alpha }\left(\tau ,\tau^{\prime }\right)}{\partial \tau^{2}}-K^{\alpha }g^{\alpha }\left(\tau ,\tau^{\prime }\right)=I\delta \left(\tau ,\tau^{\prime }\right).$$

The corresponding free GFs in frequency domain are written as:

(12) $$g_{\alpha }^{r}\left[\omega \right]={\left[{\left(\omega +i\eta \right)}^{2}-K^{\alpha }\right]}^{-1},$$

where η is an infinitesimal positive quantity to single out the correct path around the poles when performing an inverse Fourier transform, such that $g^{r}=0$ for t<0. Other Green’s functions can be obtained using the general relations among them (see Equations (2) and (3)). Hence, the contour-ordered non-equilibrium GF can be written as:

(13) $$\begin{array}{cc} G^{CL}\left(\tau ,\tau^{\prime }\right)= & \int d\tau^{\prime \prime }G^{CC}\left(\tau ,\tau^{\prime \prime }\right)V^{CL}g^{L}\left(\tau^{\prime \prime },\tau^{\prime }\right), \end{array}$$

(14) $$\begin{array}{cc} G^{CC}\left(\tau ,\tau^{\prime }\right)= & g^{C}\left(\tau ,\tau^{\prime }\right)+\int d\tau_{1}\int d\tau_{2}g^{C}\left(\tau ,\tau_{1}\right)\Sigma \left(\tau_{1},\tau_{2}\right)G^{CC}\left(\tau_{2},\tau^{\prime }\right), \end{array}$$

with $\Sigma (\tau_{1},\tau_{2})$ being the total self-energy including the coupling to the baths and given by:

(15) $$\Sigma \left(\tau_{1},\tau_{2}\right)=\Sigma_{L}\left(\tau_{1},\tau_{2}\right)+\Sigma_{R}\left(\tau_{1},\tau_{2}\right)=V^{CL}g^{L}\left(\tau_{1},\tau_{2}\right)V^{LC}+V^{CR}g^{R}\left(\tau_{1},\tau_{2}\right)V^{RC}.$$

$g^{L}$ and $g^{R}$ are the GF of the isolated semi-infinite leads. Since the bath-device interaction terms are short-ranged, it is usually only necessary to compute the projection of the bath GF on the layer directly in contact with the device. The resulting GF can be calculated by an iteration method [83] or by decimation techniques [84]. The Dyson equation (see Equation (14)) can be written in the frequency domain as:

(16) $$\begin{array}{c} \begin{array}{cc} G_{CC}^{r}\left[\omega \right]= & {\left({\left(\omega +i\eta \right)}^{2}I-K^{C}-\Sigma^{r}\left[\omega \right]\right)}^{-1},\\ G_{CC}^{<}\left[\omega \right]= & G_{C}^{r}\left[\omega \right]\Sigma^{<}\left[\omega \right]G_{C}^{a}\left[\omega \right]. \end{array} \end{array}$$

Several physical quantities can be calculated using these relations, e.g., the local density of states (LDOS) is expressed as:

(17) $$\eta_{i}\left(\omega \right)=-\frac{2\omega }{\pi }{\left(ImG^{r}\left[\omega \right]\right)}_{ii}\;,$$

and the total phonon DOS as $\eta \left(\omega \right)=\sum_{i=1}^{N}\eta_{i}\left(\omega \right)$. The DOS and LDOS give the distribution of phonons in frequency as well as in real space [81]. This is very useful to analyze quantum transport processes, as shown below. Although Equation (17) is obtained for the special case of no phonon–phonon interactions, the same formula is valid in the presence of phonon–phonon interactions described by an interaction self-energy such as in Equation (16). This typical approach assumes that the non-crossing approximation applies, allowing to treat the effect of contacts and interactions as two independent additive contributions. Clearly, this is valid in the limit of small interactions acting only within the central region.

Next, it is useful to introduce the Γ function describing the phonon scattering rate into the thermal baths:

(18) $$\Gamma \left[\omega \right]=i\left(\Sigma^{r}\left[\omega \right]-\Sigma^{a}\left[\omega \right]\right)=\Gamma_{L}\left[\omega \right]+\Gamma_{R}\left[\omega \right],$$

This function has an important relation with the spectral function, $A\left[\omega \right]=G^{r}\left[\omega \right]\Gamma \left[\omega \right]G^{a}\left[\omega \right]$. By applying the Langreth theorem [64] to Equation (13), the lesser GF $G_{CL}^{<}$ turns into:

(19) $$G_{CL}^{<}\left[\omega \right]=G_{CC}^{r}\left[\omega \right]V^{CL}g_{L}^{<}\left[\omega \right]+G_{CC}^{<}\left[\omega \right]V^{CL}g_{L}^{a}\left[\omega \right].$$

Consequently, the heat flux coming from the left lead (see Equation (9)) can be written as:

(20) $$J_{L}=-\frac{1}{2\pi }\int_{-\infty }^{+\infty }d\omega \omega \mathrm{Tr}\left(G^{r}\left[\omega \right]\Sigma_{L}^{<}\left[\omega \right]+G^{<}\left[\omega \right]\Sigma_{L}^{a}\left[\omega \right]\right).$$

For simplicity, the subscripts *C* related to the central region have been dropped. The upperletters are used to identify Green’s functions on the central region and lowercase letters for the leads. After symmetrizing with respect to the left and right leads, the heat flux becomes:

(21) $$J=\frac{1}{4}\left(J_{L}+J_{L}^{*}-J_{R}-J_{R}^{*}\right)\;.$$

The final expression reads:

(22) $$J=\int_{0}^{\infty }\frac{d\omega }{2\pi }\hbar \omega \tau_{ph}\left[\omega \right]\left(f_{L}-f_{R}\right).$$

This result is formally similar to the Landauer equation obtained for electron transport. Here, however, $f_{L,R}$ are the Bose–Einstein distributions for the left and right leads and $\tau_{ph}\left[\omega \right]$ is the phonon transmission function, given by:

(23) $$\tau_{ph}\left[\omega \right]=\mathrm{Tr}\left(G^{r}\left[\omega \right]\Gamma_{L}\left[\omega \right]G^{a}\left[\omega \right]\Gamma_{R}\left[\omega \right]\right)\;.$$

The retarded GF of the central region connected to the thermal baths is given by:

(24) $$G^{r}\left[\omega \right]={\left[{\left(\omega +i\eta \right)}^{2}I-K^{C}-\Sigma_{L}^{r}\left[\omega \right]-\Sigma_{R}^{r}\left[\omega \right]\right]}^{-1}$$

Then, the thermal conductance can then be computed according to $\kappa_{ph}=\lim_{\Delta T\to 0}\frac{J}{\Delta T}$, ΔT as the temperature difference between the thermal baths, with $T_{L}=T+\Delta T/2$ and $T_{R}=T-\Delta T/2$, respectively. A linear expansion of the Bose–Einstein distribution in ΔT yields [80]:

(25) $$\kappa_{ph}=\frac{1}{2\pi }\int_{0}^{\infty }d\omega \omega T\left[\omega \right]\frac{\partial f(\omega )}{\partial T}.$$

Notice that the thermal conductance can only be obtained by an integration over the whole frequency range of the phonon transmission. In practice, the derivative of the Bose–Einstein distribution will reduce (depending on the temperature) the real integration range. This is in contrast to the Landauer conductance for electrons, where, strictly speaking, only states near the Fermi energy are playing a role.

### 2.2. Density Functional Tight-Binding

The main quantities to obtain the quantum phonon transport properties by using the NEGF formalism are the mass-reduced force constant matrix in each region $K^{\alpha }$ (α=L,C,R) and the coupling matrices of the left and right bath to the central region, $V^{LC}$ and $V^{CR}$, respectively [80]. The accuracy of the results depends on the reliability of these quantities to catch the atomistic features of the system. Density-functional theory (DFT) is nowadays the main computational approach used in chemistry and physics to perform quantitative studies on molecules and materials due to its favorable accuracy-to-computational-time ratio [85]. The strong increase in accuracy coming from the development of gradient corrected and hybrid functionals such as PBE [86] and B3LYP [87], which compensate deficiencies of older approximations, has largely contributed to a further increase in popularity. However, hybrid functionals are computationally demanding, limiting DFT to a maximum of a few hundreds of atoms, depending on the chemical species. Classical force fields appear as a reasonable solution to this problem but, in many cases, they suffer of limited transferability and do not yield any information on the electronic structure.

Semiempirical methods appear as another option to DFT, conceptually lying between empirical force fields and first principle approaches, allowing for the treatment of thousands of atoms [88]. These methods can be understood as approximations to more accurate methods (full DFT or Hartree–Fock), but including empirical parameters that are fitted to reproduce reference data. One example of a semiempirical method, which is used in the present work, is the density functional tight-binding (DFTB) approach [89,90,91]. Here, the basic electronic parameters (Slater–Koster parameters) are consistently obtained from full DFT-based calculations for atom pairs, while the repulsive part of the electronic energy is fitted by means of splines. Based on it, the Hamiltonian and Overlap matrices of a specific system can be decomposed into pair interactions (not only between nearest-neighbors) yielding a generalized tight-binding Hamiltonian. Many studies have been carried out by using the DFTB method, including transport properties of 2D materials [92,93,94], stability and mechanical properties [95], vibrational signatures [96], computation of molecular absorption spectra [97], and of charge transfer excitation energies [98] (see recent review papers [99,100,101] for additional topics).

Three different DFTB models have been proposed up to now, which are derived by expanding the DFT total energy functional around a reference density $\rho_{0}$ to first, second, and third order, respectively [101]. The choice of the DFTB model depends on the system under study. The non self-consistent DFTB method (or DFTB0) is more appropriate for systems with negligible charge transfer between atoms (typically homonuclear systems or those involving atoms of similar electronegativity as in hydrocarbons [102]). Ionic systems with large inter-atomic charge transfer can also be treated with this method [103]. On the other hand, in systems where a delicate charge balance is crucial such as biological and organic molecules [104,105], a self-consistent charge treatment is required (DFTB2 and DFTB3) [101,106]. Based on the advantages of DFTB for accurately describing large systems involving few thousand of atoms, the force constant matrices of the studied systems are numerically obtained by applying a finite difference method to get the second derivatives of the total energy with respect to the atomic displacements (implemented in the DFTB+ software) [80]. These matrices can also be obtained by density functional perturbation theory, which in the case of DFTB reduces to analytic expressions involving derivatives of only two-center matrix elements [107].

### 2.3. Application of the DFTB-Based PHONON Tool

From electron transport studies, it is well-known that transport properties of nanoscale systems can be tailored by varying different control parameters. This can include covalent or non-covalent chemistry [108,109], atomic doping [35,110], topological defects [111,112], quantum confinement [113], and mechanical strains [114,115], among others. Similarly, a major focus of research on phonon transport is to identify the major variables allowing for effectively tuning the heat transport properties of nanoscale materials. In this section, we review few of our previous research in this direction using the NEGF-DFTB method [116,117,118,119,120,121], which is already implemented as a tool in the DFTB+ code (for details of the PHONON tool, see [80]). We focus on 2D orthorhombic materials, BNC heteronanotubes, and phonon filter effects in molecular junctions.

#### 2.3.1. 2D Orthorhombic Materials

**Figure 3:**
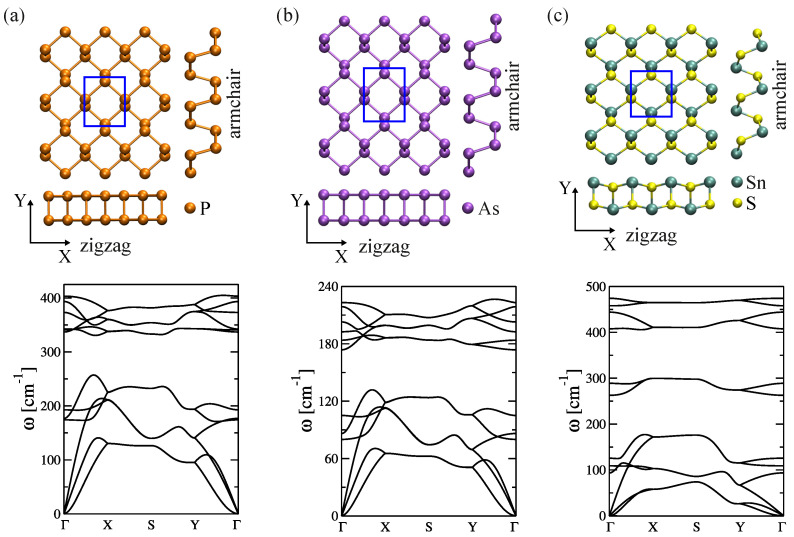
Phonon dispersion for homo-and heteroatomic two-dimensional puckered materials: (**a**) phosphorene; (**b**) arsenene; and (**c**) tin sulfide (SnS) monolayer. We also show the atomistic view of the two-dimensional materials, highlighting the zigzag (ZZ) and armchair (AC) transport directions. The figure is reproduced with permission from Ref. [117]. Copyright 2016 American Chemical Society.

The effect of anisotropic atomic structure on the phonon transport of two-dimensional puckered materials was studied by Medrano Sandonas et al. [117]. From this new family of 2D materials [122,123,124,125], three representative members, phosphorene, arsenene, and SnS monolayers, which display the main features of this family, were studied. The unit cell of these materials is composed by four atoms, as depicted in Figure 3. Each atom is pyramidally bonded to three neighboring atoms of the same type (phosphorene and arsenene, for homoatomic) or of different type (tin sulfide (SnS), for heteroatomic) forming a puckered-like honeycomb lattice. As shown in Table 1, the lattice constants computed with the DFTB approach quantitatively agree (error ≤ 5%) with those obtained at the full DFT level by other authors for all three materials.

We used a standard approach to compute the phonon band structure [80]. This consists in diagonalizing the dynamical matrix at selected k-points, after obtaining them through a Fourier transformation of the real-space force constants (this method is also part of the PHONON tool). Due to the absence of imaginary frequencies, all studied systems can be considered as mechanically stable (see the three lower panels of Figure 3). The acoustic branches display the typical dispersion of 2D materials: longitudinal (LA) and transversal (TA) acoustic branches show linear dispersion as *q* (wave vector) approaches the Γ point, while out-of-plane ZA branches show on the other hand a quadratic dispersion as a result of the rapid decay of transversal forces. The behavior of the dispersion relation for homoatomic puckered materials is almost identical, except for the maximum frequency of the optical modes, which is a consequence of the difference in mass between As (∼75 u) and P (∼31 u). We also remark that the phonon dispersion for P and As computed with DFTB agrees quite well with DFT results [117]. Only for SnS monolayer the high frequency optical modes are shifted upwards.

Furthermore, based on the group velocities values obtained for ZZ (Γ→X) and AC (Γ→Y) transport directions, we may expect that these materials will display strong anisotropy in their thermal transport. Indeed, the group velocities for the longitudinal acoustic (LA) branch in phosphorene were found to be 8.35 km/s and 4.74 km/s along the Γ-X (ZZ) and Γ-Y (AC) directions, respectively, comparable to DFT results [127,131,132]. The values for arsenene, 5.01 km/s for ZZ and 2.71 km/s for AC, are also in good agreement with those in Ref. [129]. The SnS monolayer displayed group velocities of 6.48 km/s (ZZ) and 2.14 km/s (AC). We note that thermal anisotropy has only been reported for phosphorene [126,132] and arsenene [128,129], but not for SnS monolayers. Accordingly, the largest anisotropy in the thermal conductance was found in SnS monolayers due to the dominant contribution of acoustic modes to thermal transport [117].

#### 2.3.2. Doping Influence on BNC Heteronanotubes

**Figure 4:**
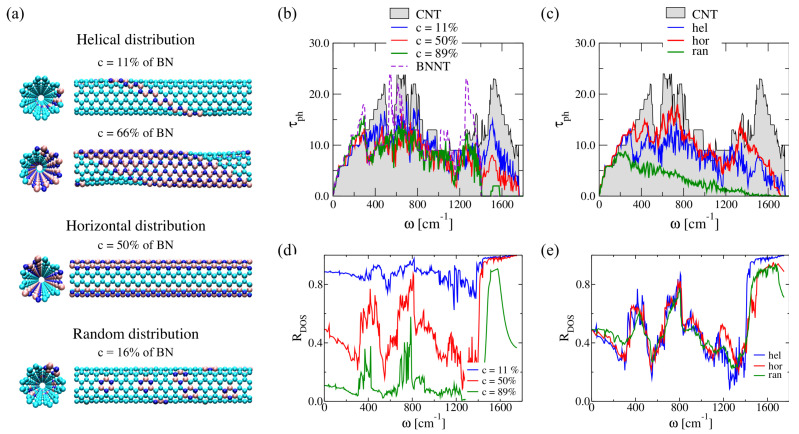
(**a**) Atomistic view of BNC heteronanotubes with helical, horizontal, and random distribution of BN domains. Carbon atoms (cyan), boron atoms (pink), and nitrogen atoms (blue) are shown. (**b**) Variation of the phonon transmission function, $\tau_{ph}$, of helical BNC heteronanotubes after increasing the BN concentration, *c*. (**c**) Comparison of $\tau_{ph}$ for different doping distribution patterns with c=50%. Variation of $R_{DOS}$ for carbon domains as a function of the vibrational frequency (**d**) for helical BNC heteronanotubes at three different doping concentrations and (**e**) for helical, horizontal, and random BNC heteronanotubes at c=50%. Reproduced from Ref. [120] with permission from the PCCP Owner Societies.

Ternary boron carbonitride nanotubes have recently been in the focus of theoretical and experimental activities because of their excellent mechanical, electrical, and non-linear optical properties which could be controlled by varying their chemical composition [133,134,135]. Hence, BNC heteronanotubes may play an important role as new generation of thermoelectric materials, and are also of great interest in environmentally relevant issues such as waste heat recovery and solid-state cooling [9,136]. In Ref. [120], we studied the influence of doping on the thermal transport properties of (6,6)-BNC heteronatubes, by considering three different BN doping distribution patterns of a carbon nanotube: helical, horizontal, and random. For this, a (6,6)-CNT of length 43.3 Å was the reference structure (supercell composed by 432 C atoms). Helical BN strips, BN chains (parallel to the transport direction, which corresponds to the z-axis), and BN rings (one ring containing 3B and 3N atoms) were introduced in an otherwise perfect (6,6)-CNT to represent helical, horizontal, and random impurity distributions (see Figure 4a). For a helical distribution, the BN concentration was varied from c=11% to c=89%, while for other cases concentrations ranging from c=16% to c=84% were studied. The limits of 0% and 100% correspond to pure carbon and hexagonal boron–nitride nanotubes, respectively.

The geometry of the BNC heteronanotubes (BNC-HNT) was optimized with the DFTB method [137,138] with periodic boundary conditions along the z-axis. C-C and B-N bond lengths amount to 1.43 Å and 1.48 Å, respectively. The optimized helical BNC-HNT presented a wave-like profile along the axial direction resulting from the difference between bond lengths at the interfaces (see, e.g., [80,139,140]). Since the doping distribution can be introduced in different ways, the phonon transmission for random and horizontal distributions were averaged over five and three different atomic configurations, respectively. For the transport calculations, the baths are composed of twice the optimized supercell, and the central region includes only one supercell. To have a better understanding of the influence of doping on the transport properties, we introduce the quantity $R_{DOS}=\eta_{X}\left(\omega \right)/\eta_{Total}\left(\omega \right)$, where $\eta_{Total}\left(\omega \right)$ is the total DOS given by Equation (17), and $\eta_{X}\left(\omega \right)$ can be either the LDOS of C or BN domains.

In Figure 4b, the influence of helical BN stripes on the phonon transmission of a (6,6)-CNT is shown. The high frequency modes ($\omega >1400\;{\mathrm{cm}}^{-1}$) are strongly affected by increasing the BN concentration. These modes correspond to local vibrations related to carbon atoms; this is seen in $R_{DOS}$ after increasing the doping concentration (see Figure 4d). On the contrary, the transmission of low-frequency vibrations below 200 ${\mathrm{cm}}^{-1}$ is not changed much when varying the disorder concentration. Figure 4c shows the phonon transmission of BNC-HNT with fixed concentration c=50% and different BN spatial arrangements. As expected, a random distribution of B and N atoms blocks the transmission over almost the whole frequency spectrum; only low-frequency modes experience less scattering at the localized impurities, so that their transmission is much less affected. Helical and horizontal disorder in BNC-HNT leads to a stronger blocking of the transmission at high frequencies ($\omega >1400\;{\mathrm{cm}}^{-1}$) due to the absence of B-N-C local vibrations in that range (see Figure 4e).

**Figure 5:**
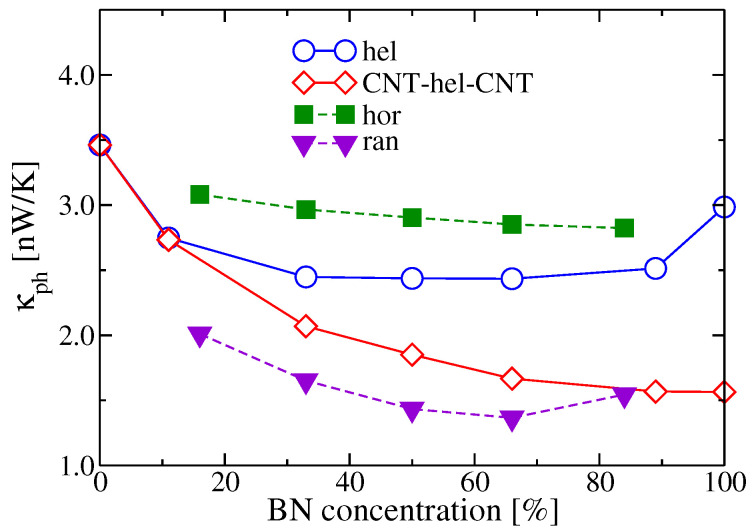
Phonon thermal conductance as a function of the BN concentration for helical, horizontal, and random pattern distributions. Results for helical BNC heteronanotubes connected to two CNT leads are also shown. Reproduced from Ref. [120] with permission from the PCCP Owner Societies.

Figure 5 shows the concentration dependence of the phonon thermal conductances, $\kappa_{ph}$, for each doping distribution pattern at T=300 K. Horizontal BNC-HNT shows the highest thermal conductance, while the lowest $\kappa_{ph}$ is obtained for (6,6)-CNT with BN domains randomly distributed. The thermal conductance of helical BNC-HNT remains nearly constant (∼2.5 nW/K) for concentrations between 30% and 80%, and then increases until it reaches the value corresponding to a pristine BNNT, ∼3.0 nW/K. An additional case was studied with a helical BNC-HNT connected to CNT leads and, as a consequence of the new contact-device interface, the thermal conductance is continuously suppressed with increasing concentration. Notice that the dominant contribution to the thermal conductance at 300 K mostly derives from long wavelength modes with frequencies $\leq 200\;{\mathrm{cm}}^{-1}$ [120].

#### 2.3.3. Selective Molecular-Scale Phonon Filtering

**Figure 6:**
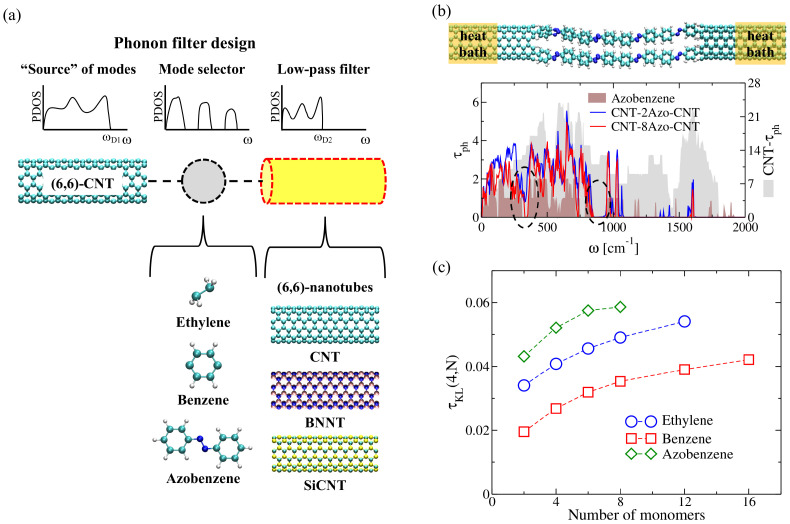
(**a**) Schematic representation of the nanoscale phonon filter proposed in Ref. [121]. A two-terminal junction is considered, where the role of the thermal baths is played by two semi-infinite (6,6) nanotubes (CNT, BNT, SiCNT) which are bridged by molecular chains consisting of ethylene, benzene, and azobenzene monomers. $\omega_{D}$ represents the Debye frequency in each nanotube. (**b**) Phonon transmission functions $\tau_{ph}$ for benzene-based junctions. We also added the plot corresponding to the phonon transmission function of and infinite CNT (grey) and a single infinite molecular chain of benzene monomers (brown). Highlighted with dashed-line circles are the regions where phonon gaps clearly develop by increasing the chain length. (**c**) Variation of $\tau_{KL}\left(j=4,N\right)$ as a function of the number of monomers (*N*) in the different studied molecular junctions by considering both thermal baths made of (6,6)-CNTs. Each junction consists of four molecular chains in parallel, j=4. The figure is reproduced with permission from Ref. [121]. Copyright 2019 American Chemical Society.

We have recently proposed nano-junctions consisting of two colinear (6,6)-nanotubes (NT) joined by a central molecular structure as a potential molecular-scale phonon filter (see Figure 6a) [121]. The nanotubes act as heat baths kept at the same temperature. The left NT is considered as a reference bath with a broad phonon frequency spectrum, playing the role of a “source” of phonon modes. The molecular system in the central part is as a mode selector, and selected modes are then propagated to the right contact [80]. Besides carbon NTs, boron-nitride (BN) and silicon carbide (SiC) nanotubes as right baths were also considered. The filtering capability of the device thus depends on a mode-specific propagation resulting from the combined effect of molecular vibrations selection rules and the overlap of the contact spectral densities with the molecular region.

**Figure 7:**
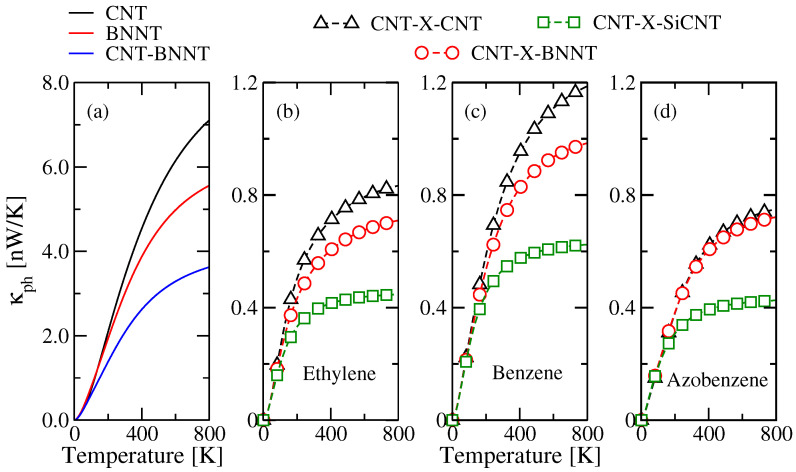
Phonon thermal conductance $\kappa_{ph}$ as a function of the temperature. (**a**) $\kappa_{ph}$ values of the infinite CNT and BNNT as well as of the CNT-BNNT junctions. The remaining panels show the thermal conductance in the different junction types with a chain length corresponding to four monomers for: (**b**) ethylene; (**c**) benzene; and (**d**) azobenzene. The figure is reproduced with permission from Ref. [121]. Copyright 2019 American Chemical Society.

The phonon transport problem was treated using the previously described Green’s function technique as implemented in the PHONON tool. Figure 6b,c shows the influence of interconnecting chains on the filtering effect for the case where both thermal baths are (6,6)-CNTs with $\omega_{D}∼1800$ cm${}^{-1}$. As bridging molecular systems four parallel chains of ethylene, benzene, and azobenzene were chosen [121]. In Figure 6b, spectral gaps emerge in the transmission induced by the presence of the molecular chains. The overall transmission of the junctions is reduced by roughly a factor of four when compared with the infinite CNT due additional phonon scattering effects at the interfaces. The influence of azobenzene chains is stronger comparing to the other monomers. Thus, chains with only two monomers already induce phonon gaps and filter out roughly half of the spectral range (see the lower panel of Figure 6b). Contrary to the benzene case, the transmission at low frequencies was strongly reduced, a result probably related to the lower number of modes and additional scattering at lower frequencies induced by larger structural distortions [141,142]. As a result, azobenzene-based junctions display the lowest thermal conductance, $\kappa_{ph}$ (see Figure 7b for only CNT-based leads). In brief, it becomes clear that channel selection and phonon filtering can be strongly controlled by the chemical composition of the bridge [121].

To quantify the deviation from the “source of modes” distribution (or degree of filtering), quantified in our case by the transmission spectrum of the CNT, a key design magnitude $\tau_{\mathrm{KL}}\left(j,N\right)$ was defined as [121]:

(26) $$\tau_{\mathrm{KL}}\left(j,N\right)=\frac{1}{\omega_{D}^{\mathrm{CNT}}}\int_{0}^{\omega_{D}^{\mathrm{CNT}}}d\omega \;\tau_{\mathrm{CNT}}\left(\omega \right)\;\ln\frac{\tau_{\mathrm{CNT}}\left(\omega \right)}{\tau_{\mathrm{MJ},j}\left(\omega \right)},$$

with the index *j* referring to the number of molecular chains in parallel interconnecting the two thermal baths and *N* the number of monomers in one chain. $\tau_{\mathrm{CNT}}\left(\omega \right)$ and $\tau_{\mathrm{MJ},j}\left(\omega \right)$ are the corresponding transmission functions for an infinite CNT and for the CNT-molecule junction containing *j* molecular chains in parallel. A perfect filter (zero transmission) yields $\tau_{\mathrm{KL}}\left(j,N\right)\to \infty$, while no filtering at all yields $\tau_{\mathrm{KL}}\left(j,N\right)=0$. As shown in Figure 6c, the azobenzene junctions display the highest $\tau_{\mathrm{KL}}$ (i.e., highest filter efficiency) due to the efficient blocking of high-frequency modes above 1000 cm${}^{-1}$. The ethylene-based chains are less efficient, but still display a larger effect than the benzene chains; this is mostly related to two issues: the complete filtering of frequencies larger than 1500 cm${}^{-1}$ and the presence of a relatively large phonon gap between 500 cm${}^{-1}$ and 750 cm${}^{-1}$. $\tau_{\mathrm{KL}}$ for benzene-based junctions also increases with the length of the molecular chain and shows a tendency to saturate for N>12 monomers.

Figure 7 highlights the influence of changing the material of the right bath on the phonon filtering. In these calculations, all chains consist of four monomers. The thermal conductance of CNT-BNNT junctions is reduced when compared to the perfect tubes due to interface scattering (see Figure 7a [143,144,145,146]). By inserting the molecular system, a reduction of the thermal conductance by roughly a factor of 3–4 is produced, the effect being more pronounced for the azobenzene junction [121]. For azobenzene-based molecular junctions, the thermal conductance barely changes when going from CNT-CNT to CNT-BNNT (see Figure 7d). This is a consequence of the strong suppression of high frequency transport channels in the transmission. The saturation of the thermal conductance is determined by the nanotube with the smaller Debye cutoff (going from CNT ($\omega_{D}∼1800$ cm${}^{-1}$) to BNNT ($\omega_{D}∼1400$ cm${}^{-1}$) to SiCNT ($\omega_{D}∼880$ cm${}^{-1}$)), the value of the saturation point is, however, influenced by the specific composition of the molecular chains.

## 3. Atomistic Framework for Time-Dependent Thermal Transport

A novel atomistic approach able to treat transient phonon transport was recently developed by Medrano Sandonas et al. [147]. The approach is based on the solution of the equation of motion for the phonon density matrix σ(t), calculated within the NEGF formalism, by using an auxiliary-mode approach [80,147]. The latter has been previously used for time-resolved electron transport [55,56,57]. Unlike recent related approaches with limited application range (in terms of an atomistic treatment of the underlying system) [61,62,63], our method can be efficiently combined with an atomistic description as implemented in standard first-principle or parameterized approaches.

**Figure 8:**
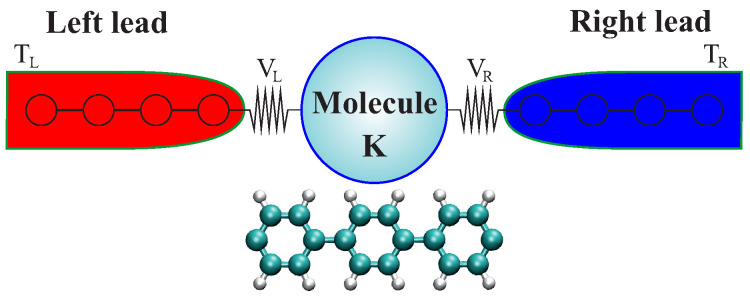
Schematic representation of the target molecular junctions by using the TD-NEGF approach. A molecular system is connected to two harmonic thermal baths, which are the source for the heat flow in the molecule. The figure is reproduced with permission from Ref. [147]. Copyright 2018 American Chemical Society.

The basic structure of this approach is shown in Figure 8. Two thermal baths made of non-interacting harmonic oscillators in thermal equilibrium are contacted to a scattering region, whose vibrational features are assumed to be well represented by a quadratic Hamilton operator. The total system is described by the Hamiltonian:

(27) $$H=H_{C}+\sum_{\alpha k}\left(\frac{1}{2}p_{\alpha k}^{2}+\frac{1}{2}\omega_{\alpha k}^{2}u_{\alpha k}^{2}\right)+\sum_{\alpha ,k}\frac{1}{2}\left(u^{T}\cdot V_{\alpha k}u_{\alpha k}+u_{\alpha k}V_{\alpha k}^{T}\cdot u\right)\;.$$

The first term $H_{C}=\left(1/2\right)p^{T}\cdot p+\left(1/2\right)u^{T}\cdot K_{\mathrm{eff}}\cdot u$ is the Hamiltonian of the central domain, u is a column vector consisting of all the displacement variables in the region, and p contains the corresponding momenta. Both vectors have length *N*, with *N* being the number of degrees of freedom in the central region. We chose renormalized displacements $u_{i}=\sqrt{m_{i}}x_{i}$, where $m_{i}$ is the mass associated to the *i*th vibrational degree of freedom, and $x_{i}$ is the actual displacement having the dimension of length [80,147]. The effective force-constant matrix $K_{\mathrm{eff}}=K+K_{\mathrm{ct}}$ has dimension N×N, and includes the force constant matrix of the central region, K, and a counter-term $K_{\mathrm{ct}}$ [80,147]. The index α∈{L,R} labels the left (L) and right (R) heat baths and *k* denotes their vibrational modes with frequency $\omega_{\alpha k}$. The second term of Equation (27) is the Hamiltonian of the heat bath. The last term represents the interaction between the central region and the baths, given by coupling vectors $V_{\alpha k}$ which are assumed to vanish before time $t_{0}\to -\infty$. Written in this form, the coupling leads to a renormalization of the bare force-constant matrix, which can be canceled by the above introduced counterterm $K_{\mathrm{ct}}=\sum_{\alpha ,k}\left(V_{\alpha k}\cdot V_{\alpha k}^{T}\right)/\omega_{\alpha k}^{2}$ [80,147]. Hence, the coupling to the thermal baths will introduce dissipation but no shift in the vibrational spectrum [148]. The equations of motion (EOM) for the central region (u and p) and normal modes of one lead ($u_{k}$) read [80,147]:

(28) $$\frac{\partial }{\partial t}\left(\begin{array}{c} u\\ p \end{array}\right)=K_{\mathrm{eff}}\cdot \left(\begin{array}{c} u\\ p \end{array}\right)-\sum_{k}u_{k}\left(\begin{array}{c} 0\\ V_{k} \end{array}\right).$$

The 2N×2N dimensional auxiliary matrices (denoted by caligraphic symbols) are given by the expressions [80,147]:

$$I\equiv \left(\begin{array}{cc} I & 0\\ 0 & I \end{array}\right),\;Q\equiv \left(\begin{array}{cc} 0 & I\\ -I & 0 \end{array}\right),\;K_{\mathrm{eff}}\equiv \left(\begin{array}{cc} 0 & I\\ -K_{\mathrm{eff}} & 0 \end{array}\right)\equiv \left(\begin{array}{cc} 0 & I\\ -K & 0 \end{array}\right)+\left(\begin{array}{cc} 0 & 0\\ -K_{\mathrm{ct}} & 0 \end{array}\right).$$

The energy of the central region can be written in terms of the phonon density matrix $\sigma \left(t\right)=iG^{<}\left(t,t\right)$, with $G^{<}\left(t,t\right)$ being a lesser Green’s function [80,147]:

(29) $$\begin{array}{c} E_{C}\left(t\right)=\frac{1}{2}\mathrm{Tr}\;\left\{K_{\mathrm{eff}}^{T}\cdot Q\cdot \sigma \left(t\right)\right\}, \end{array}$$

and the total energy is $E_{Tot}\left(t\right)=E_{C}\left(t\right)+E_{bath}\left(t\right)$. In the absence of external forces, the total energy is conserved, and the heat current coming from the heat baths can be defined as $J\left(t\right)=-\frac{\partial }{\partial t}E_{C}\left(t\right)$. Hereafter, ℏ=1 is taken. The time evolution of the heat flux is related to the lesser GF as [62,63]:

(30) $$G^{<}\left(t,t^{\prime }\right)=-i\left(\begin{array}{cc} \langle u\left(t^{\prime }\right)u^{T}\left(t\right)\rangle & \langle u\left(t^{\prime }\right)p^{T}\left(t\right)\rangle \\ \langle p\left(t^{\prime }\right)u^{T}\left(t\right)\rangle & \langle p\left(t^{\prime }\right)p^{T}\left(t\right)\rangle \end{array}\right).$$

To obtain the time dependence of the lesser GF, Dyson’s equation was derived (see Ref. [80] for details). Using Langreth’s rules, the lesser GF can be written in terms of retarded and advanced GFs:

(31) $$\begin{array}{c} G^{<}\left(t,t^{\prime }\right)=\int d\tau_{2}\int d\tau_{3}\;G^{R}\left(t,\tau_{2}\right)\cdot S^{<}\left(\tau_{2},\tau_{3}\right)\cdot G^{A}\left(\tau_{3},t^{\prime }\right). \end{array}$$

For the latter, EOMs are given by [80,147]:

(32) $$\begin{array}{cc} \frac{\partial }{\partial t}G^{R}\left(t,t^{\prime }\right)= & \delta \left(t,t^{\prime }\right)Q+K_{\mathrm{eff}}\cdot G^{R}\left(t,t^{\prime }\right)+Q\cdot \int dt_{2}\;S^{R}\left(t,t_{2}\right)\cdot G^{R}\left(t_{2},t^{\prime }\right). \end{array}$$

(33) $$\begin{array}{cc} \frac{\partial }{\partial t^{\prime }}G^{A}\left(t,t^{\prime }\right)= & \delta \left(t,t^{\prime }\right)Q^{T}+G^{A}\left(t,t^{\prime }\right)\cdot K_{\mathrm{eff}}^{T}+\int dt_{2}\;G^{A}\left(t,t_{2}\right)\cdot S^{A}\left(t_{2},t^{\prime }\right)\cdot Q^{T}. \end{array}$$

$S^{R,A,<}$ denotes the respective self-energy. Setting $t^{\prime }\to t$ in Equation (31), the EOM for lesser GF becomes:

(34) $$\begin{array}{cc} \frac{\partial }{\partial t}G^{<}\left(t,t\right)= & K_{\mathrm{eff}}\cdot G^{<}\left(t,t\right)+G^{<}\left(t,t\right)\cdot K_{\mathrm{eff}}^{T}\\ & +Q\cdot \left[\int_{\tau_{2}}^{t}d\tau_{2}\;\left(S^{>}\left(t,\tau_{2}\right)G^{<}\left(\tau_{2},t\right)-S^{<}\left(t,\tau_{2}\right)G^{>}\left(\tau_{2},t\right)\right)\right]\\ & +\left[\int_{\tau_{2}}^{t}d\tau_{2}\;\left(G^{>}\left(t,\tau_{2}\right)S^{<}\left(\tau_{2},t\right)-G^{<}\left(t,\tau_{2}\right)S^{>}\left(\tau_{2},t\right)\right)\right]\cdot Q^{T}, \end{array}$$

where the thermal current matrices $\Pi_{\alpha }\left(t\right)$ are defined as:

(35) $$\Pi_{\alpha }\left(t\right)=\int_{\tau_{2}}^{t}d\tau_{2}\;\left(G^{>}\left(t,\tau_{2}\right)S_{\alpha }^{<}\left(\tau_{2},t\right)-G^{<}\left(t,\tau_{2}\right)S_{\alpha }^{>}\left(\tau_{2},t\right)\right).$$

For harmonic baths, $S_{\alpha }^{<,>}\left(t,t^{\prime }\right)$ can be obtained as:

(36) $$S_{\alpha }^{≶}\left(t,t^{\prime }\right)=-i\int_{0}^{\infty }\frac{d\omega }{\pi }\left[\coth\frac{\omega }{2k_{B}T_{\alpha }}\cos\omega \left(t-t^{\prime }\right)\pm i\sin\omega \left(t-t^{\prime }\right)\right]L_{\alpha }\left(\omega \right).$$

$T_{\alpha }$ is the bath temperature and $L_{\alpha }\left(\omega \right)$ is the spectral density of reservoir α [148,149]. The EOM for the phonon density matrix reads [80,147]:

(37) $$\begin{array}{c} \frac{\partial }{\partial t}\sigma \left(t\right)=K_{\mathrm{eff}}\cdot \sigma \left(t\right)+\sigma \left(t\right)\cdot K_{\mathrm{eff}}^{T}+i\sum_{\alpha \in \{L,R\}}\left(\Pi_{\alpha }\left(t\right)\cdot Q^{T}-h.c.\right). \end{array}$$

### 3.1. Auxiliary-Mode Approach

An auxiliary-mode approach was used to expand the self-energies $S_{\alpha }^{<,>}\left(t,t\right)$ in exponential functions to achieve a numerically efficient implementation to calculate the time evolution of $\Pi_{\alpha }\left(t\right)$. The approach has been previously implemented for electrons [55,56,57] and for vibrations [150,151]. Since the cos and sin functions in Equation (36) can be easily expressed in terms of exponential functions, the only term which has to be considered is the hyperbolic cotangent. Different schemes have been proposed to obtain a suitable pole decomposition of this term [150]. To bypass the slow convergence of the so-called Matsubara decomposition, a more advanced pole decomposition was suggested by Croy and Saalmann [55], based on a partial fraction decomposition method that displays a faster convergence. However, one needs in this method high-precision arithmetic to compute the poles correctly, so that it is of advantage to have a pole decomposition of the coth function with purely imaginary poles [80]. A Pade decomposition method was recently proposed by Hu et al. [152], which shows very rapid convergence: the hyperbolic cotangent can be written in terms of simple poles as [150]:

(38) $$\coth\left(x\right)\approx \frac{1}{x}+\sum_{p=1}^{N_{P}}\eta_{p}\left(\frac{1}{x-\xi_{p}}+\frac{1}{x-\xi_{p}^{*}}\right),$$

where $\eta_{p}$ are residues and $\xi_{p}$ (with Imξp>0) are the poles. Here, $N_{P}$ denotes the number of poles.

Following this auxiliary-mode approach, a spectral density with a Drude regularization (i.e., adding a cut-off frequency $\omega_{c}$) of the form $L_{\alpha }\left(\omega \right)=\left(\omega_{c}^{2}\omega \right)/\left(\omega^{2}+\omega_{c}^{2}\right)L_{\alpha }^{\left(0\right)}$ was considered [148]. $L_{\alpha }^{\left(0\right)}\equiv \mathrm{diag}\left(\Lambda_{\alpha }^{\left(0\right)},0\right)$ contains the coupling matrix $\Lambda_{\alpha }^{\left(0\right)}$ between the central domain and the leads. This quantity contains the wide-band limit as a special case for $\omega_{c}\to \infty$. Using a linear combination of Lorentzians, any spectral density can in principle be approximated. The Drude spectral density is inserted into the expression for the lesser/greater self-energies (see Equation (36)), and for $\tau =t-t^{\prime }>0$, it turns into [80,147]:

(39) $$\begin{array}{c} S_{\alpha }^{≶}\left(t,t^{\prime }\right)=L_{\alpha }^{\left(0\right)}\left[C_{\alpha }\left(\tau \right)\pm sgn\left(\tau \right)\frac{\omega_{c}^{2}}{2}e^{-\omega_{c}\left|\tau \right|}\right]. \end{array}$$

with:

(40) $$\begin{array}{c} C_{\alpha }\left(\tau \right)=-i\int_{-\infty }^{\infty }\frac{d\omega }{2\pi }\left(\frac{\omega_{c}^{2}\omega }{\omega^{2}+\omega_{c}^{2}}\right)\coth\frac{\omega }{2k_{B}T_{\alpha }}e^{i\omega \tau }, \end{array}$$

As mentioned above, the goal of the auxiliary-mode approach is to expand the self-energies and $C_{\alpha }\left(\tau \right)$ in terms of exponentials. Using the Pade decomposition of the hyperbolic cotangent in Equation (40), one obtains [80,147]:

$$\begin{array}{c} C_{\alpha }\left(\tau \right)=-ik_{B}T_{\alpha }\omega_{c}e^{-\omega_{c}\tau }-i\sum_{p=1}^{N_{P}}R_{\alpha ,p}\left(\omega_{c}e^{-\omega_{c}\tau }-\chi_{\alpha ,p}e^{-\chi_{\alpha ,p}\tau }\right), \end{array}$$

with $R_{\alpha ,p}=\frac{2k_{B}T_{\alpha }\omega_{c}^{2}}{\omega_{c}^{2}-\chi_{\alpha ,p}^{2}}\eta_{p}$ and $\chi_{\alpha ,p}=-i2k_{B}T_{\alpha }\xi_{p}$. Hence:

(41) $$\begin{array}{c} S_{\alpha }^{<,>}\left(t,t^{\prime }\right)=\frac{\partial }{\partial t}\sum_{p=0}^{N_{P}}a_{\alpha ,p}^{<,>}e^{-b_{\alpha ,p}\left(t-t^{\prime }\right)}L_{\alpha }^{\left(0\right)}=\frac{\partial }{\partial t}N^{<,>}\left(t,t^{\prime }\right) \end{array}$$

where the coefficients $a_{\alpha ,p}^{<,>}$ and $b_{\alpha ,p}$ are related to the auxiliary-mode decomposition. For τ<0, the coefficients are $a_{\alpha ,p}^{*,<,>}$ and $b_{\alpha ,p}^{*}$ [147].

The phonon current matrices $\Pi_{\alpha }\left(t\right)$ (see Equation (35)) can be written as [80,147]:

(42) $$\Pi_{\alpha }\left(t\right)=\sum_{p=0}^{N_{P}}\left[\Phi_{\alpha }^{p}\left(t\right)+a_{\alpha ,p}^{*,<}Q\cdot L_{\alpha }^{\left(0\right)}\right],$$

with:

$$\Phi_{\alpha }^{p}\left(t\right)=\int_{t_{0}}^{t}dt^{\prime }\;\left(G^{<}\left(t,t^{\prime }\right)\cdot K_{\mathrm{eff}}^{T}\cdot N_{\alpha ,p}^{>}\left(t^{\prime },t\right)-G^{>}\left(t,t^{\prime }\right)\cdot K_{\mathrm{eff}}^{T}\cdot N_{\alpha ,p}^{<}\left(t^{\prime },t\right)\right),$$

The EOM for $\Phi_{\alpha }^{p}\left(t\right)$ is given by [80,147]:

(43) $$\begin{array}{cc} \frac{\partial }{\partial t}\Phi_{\alpha }^{p}\left(t\right)= & A_{\alpha }^{p}\cdot \Phi_{\alpha }^{p}\left(t,t\right)-B_{\alpha }^{p}\cdot K_{\mathrm{eff}}^{T}\cdot L_{\alpha }^{\left(0\right)}+Q\cdot \Omega_{\alpha }^{p}\left(t\right), \end{array}$$

with $A_{\alpha }^{p}=K-b_{\alpha ,p}^{*}I$, $B_{\alpha }^{p}=i\omega_{c}\delta_{p,0}\sigma \left(t\right)+a_{\alpha ,p}^{*,<}Q$. The functions $\Omega_{\alpha }^{p}\left(t\right)=\sum_{\alpha^{\prime }}\sum_{p^{\prime }=0}^{N_{P}}\Omega_{\alpha^{\prime }\alpha }^{p^{\prime }p}\left(t\right)$ correlate the main features of both heat baths and their values are obtained by performing the time derivative of $\Omega_{\alpha^{\prime }\alpha }^{p^{\prime }p}\left(t\right)$, which is expressed as [147]:

(44) $$\begin{array}{cc} \frac{\partial }{\partial t}\Omega_{\alpha^{\prime }\alpha }^{p^{\prime }p}\left(t\right)= & C_{\alpha^{\prime }\alpha }^{p^{\prime }p}L_{\alpha^{\prime }}^{\left(0\right)}\cdot Q\cdot K_{\mathrm{eff}}^{T}\cdot L_{\alpha }^{\left(0\right)}-D_{\alpha^{\prime }\alpha }^{p^{\prime }p}\Omega_{\alpha^{\prime }\alpha }^{p^{\prime }p}\left(t\right)\\ & -\omega_{c}\left(\delta_{p^{\prime },0}L_{\alpha^{\prime }}^{\left(0\right)}\cdot K_{\mathrm{eff}}\cdot \Phi_{\alpha }^{p}\left(t\right)-\delta_{p,0}\Phi_{\alpha^{\prime }}^{p^{\prime },†}\left(t\right)\cdot K_{\mathrm{eff}}^{T}\cdot L_{\alpha }^{\left(0\right)}\right), \end{array}$$

where $C_{\alpha^{\prime }\alpha }^{p^{\prime }p}=a_{\alpha^{\prime },p^{\prime }}^{<}a_{\alpha ,p}^{*,>}-a_{\alpha^{\prime },p^{\prime }}^{>}a_{\alpha ,p}^{*,<}$ and $D_{\alpha^{\prime }\alpha }^{p^{\prime }p}=b_{\alpha^{\prime },p^{\prime }}+b_{\alpha ,p}^{*}$ are real numbers. Thus, the initial integro-differential equation for the reduced density matrix has been mapped onto a closed set of ordinary differential equations (Equations (37), (43), and (44)), which can be solved using, e.g., a fourth-order Runge–Kutta method [80,147].

The thermal current is calculated as [80,147]:

(45) $$\begin{array}{c} J\left(t\right)=-\frac{i}{2}\mathrm{Tr}\left\{K_{\mathrm{eff}}^{T}\cdot Q\cdot \sum_{\alpha }\left[\Pi_{\alpha }\left(t\right)\cdot Q^{T}-h.c.\right]\right\}. \end{array}$$

### 3.2. Applications of TD-NEGF Approach

#### 3.2.1. Proof-of-Principle: One-Dimensional Atomic Chain

**Figure 9:**
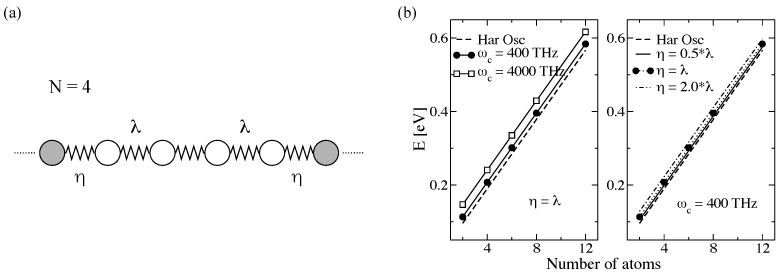
(**a**) Scheme of the one-dimensional atomic chain studied in this work for the case of N=4 atoms, with the filled atoms representing the beginning of the heat baths. λ and η are the spring force constants between the atoms and the coupling of the central region to the baths. Time-dependent NEGF approach: (**b**) Variation of the total energy of a dimer at $T_{0}=300$ K after increasing the number of atoms in the one-dimensional atomic chain for different cut-off frequency (**left**) and η parameter (**right**). For comparison, we also plotted the energy values corresponding to the ideal harmonic oscillator case (dashed lines). Panel (**b**) is reproduced with permission from Ref. [147]. Copyright 2018 American Chemical Society.

To illustrate this approach, a one-dimensional (1D) chain with *N* atoms interacting via force constants with strength λ= 1.0 eV/μÅ was considered (see Figure 9a) for N=4 [80,147]. The atomic masses of the atoms was set to 1.0 μ, and only nearest-neighbor interactions were considered.

First, a benchmark of the influence of spectral density parameters on the steady state properties was carried out. In Figure 9b, the dependence of the system energy at 300 K on *N* for different cut-off frequency $\omega_{c}$ and η parameter is displayed [80,147]. The energy is compared to that obtained for an ideal harmonic oscillator in equilibrium, $E_{C}=\sum_{i=1}^{N}\hbar \omega_{i}\left(n(\omega_{i})+1/2\right)$, with n(ω) being the Bose–Einstein distribution function and $\omega_{i}$ are the frequencies of the isolated central system. For fixed η=λ, increasing $\omega_{c}$ leads to an increase of the system energy, since the spectral density includes more vibrational states. Contrarily, the energy gets closer to the harmonic oscillator values after reducing η for fixed $\omega_{c}=400$ THz. This phenomena is expected because, for η→0, the chain can be considered as an isolated harmonic system with no heat injection from the bath and $E_{1\mathrm{Dchain}}=E_{C}$.

A similar effect is also found by analyzing the phonon transmission $\tau_{ph}\left(\omega \right)$ calculated along the lines of Section 2.1. Using the spectral density $\Lambda \left(\omega \right)=\left(\omega_{c}^{2}\omega \right)/\left(\omega^{2}+\omega_{c}^{2}\right)\Lambda^{\left(0\right)}$, one gets [147]:

(46) $$\begin{array}{cc} \Sigma_{\alpha }^{≶}\left(t,t^{\prime }\right)= & -i\int_{-\infty }^{\infty }\frac{d\omega }{2\pi }\left(\frac{\omega_{c}^{2}\omega }{\omega^{2}+\omega_{c}^{2}}\right)\Lambda_{\alpha }^{\left(0\right)}\left[\coth\frac{\omega }{2k_{B}T_{\alpha }}\pm 1\right]e^{i\omega (t-t^{\prime })}. \end{array}$$

Hence, the retarded self-energy in time domain is written as:

(47) $$\begin{array}{c} \Sigma_{\alpha }^{r}\left(t,t^{\prime }\right)=\Theta \left(t-t^{\prime }\right)2i\Lambda_{\alpha }^{\left(0\right)}\int_{-\infty }^{\infty }\frac{d\omega }{2\pi }\left(\frac{\omega_{c}^{2}\omega }{\omega^{2}+\omega_{c}^{2}}\right)e^{i\omega (t-t^{\prime })}. \end{array}$$

By performing a Fourier transform of the previous equation and considering the counter-term, the self-energies are given by:

(48) $$\Sigma_{\alpha }^{r}\left(\omega \right)=\left[i\frac{\omega_{c}^{2}\omega }{\omega^{2}+\omega_{c}^{2}}\right]\Lambda_{\alpha }^{\left(0\right)}={\left[\Sigma_{\alpha }^{a}\left(\omega \right)\right]}^{†}.$$

The retarded Green-function finally reads:

(49) $$G^{r}\left(\omega \right)={\left[\omega^{2}I-K-\Sigma_{L}^{r}\left(\omega \right)-\Sigma_{R}^{r}\left(\omega \right)\right]}^{-1},$$

where K is the force constant matrix of the one-dimensional chain. The transmission function $\tau_{ph}\left(\omega \right)$ is computed with Equation (23) and the steady heat flux is calculated by using the Landauer approach (see Equation (22)).

**Figure 10:**
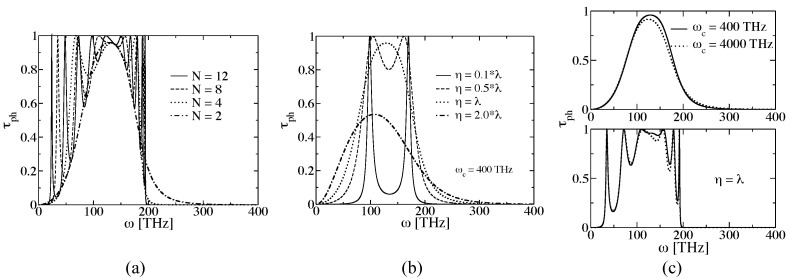
Landauer approach: (**a**) Variation of the phonon transmission function $\tau_{ph}$ of an one-dimensional atomic chain as a function of the number of atoms. (**b**) Influence of the coupling parameter on the phonon transmission function $\tau_{ph}$ of an atomic dimer. (**c**) Variation of $\tau_{ph}$ with respect to the cut-off frequency for N=2 (**top**) and N=4 (**bottom**). The figure is reproduced with permission from Ref. [147]. Copyright 2018 American Chemical Society.

Figure 10a shows $\tau_{ph}\left(\omega \right)$ for different *N* with a cut-off at 400 THz. New transmission peaks appear for larger *N* due to emergence of new vibrational modes [80]. In addition, it was found that the maximum frequency $\omega_{max}$ with non-zero transmission depends on *N*. Thus, for N>8, $\omega_{max}$ remains constant (∼195 THz). For N→∞, $\tau_{ph}$ is constant and is zero for $\omega >\omega_{max}$, i.e., all modes have the same transmission probability $\tau_{ph}=1.0$ (see also [33]). Figure 10b shows the influence of η for a dimer with $\omega_{c}=400$ THz. η has a considerable influence on the phonon transmission. For η≥λ, $\tau_{ph}$ is similar to a Gaussian and the dimer cannot be understood as a weakly coupled system anymore. For η≤0.8λ, on the contrary, two main transmission peaks corresponding to the two dimer modes can be resolved. For even weaker coupling, $\tau_{ph}$ will yield two delta functions at these frequencies, correctly describing the vibrations of the system. This result confirms the analysis carried out for the system energy and shown in Figure 9b. The influence of the cut-off frequency on $\tau_{ph}$ is weak compared to η: increasing $\omega_{c}$ ten times only leads to a slight reduction of the phonon transmission at high frequencies and the frequency spectrum becomes wider (see Figure 10c) [80,147].

**Figure 11:**
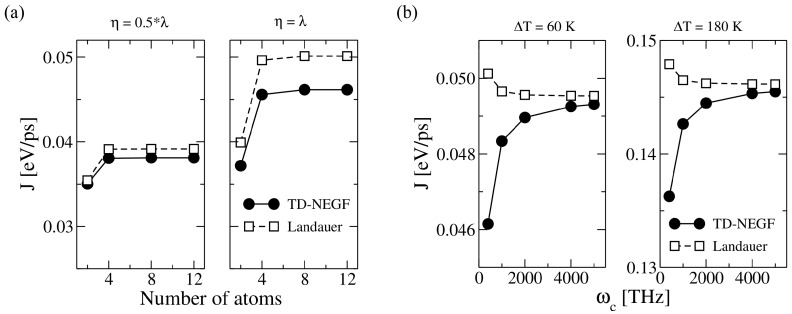
Landauer approach: (**a**) Steady heat flux as a function of the number of atoms in the one-dimensional atomic chain for different η values. (**b**) Cut-off frequency $\omega_{c}$ dependence of the steady heat flux for the atomic dimer at various temperatures bias ΔT. For comparison, we also plotted the values obtained using Landauer approach. The figure is reproduced with permission from Ref. [147]. Copyright 2018 American Chemical Society.

Based on the Landauer formalism, the temperature of the heat baths only appears in the Bose–Einstein distribution (see Equation (22)). However, in the TD-NEGF approach, the temperatures appear in the auxiliary-mode expansion of the self-energy. Once the system is in thermal equilibrium, a symmetric temperature bias $\Delta T=T_{L}-T_{R}=2\xi T_{0}$ (ξ>0) is applied, with $T_{0}$ being the mean temperature at which the system was previously equilibrated [80,147]. The left and right baths temperatures are expressed as $T_{L}=\left(1+\xi \right)T_{0}$ and $T_{R}=\left(1-\xi \right)T_{0}$. Figure 11a shows the steady heat flux as a function of *N* for different η ($\omega_{c}=400$ THz). The heat flux values were obtained for a mean temperature of $T_{0}=300$ K and ξ=0.1. The values in both methods become closer after reducing η in agreement with the above discussed results in Figure 9b and Figure 10b. Additionally, the heat flux converges for a given *N* for all ηs [80,147].

The influence of $\omega_{c}$ on the steady heat flux was studied for η=λ (see Figure 11b). One sees that each approach displays a different behavior, i.e., the heat flux increases and decreases after increasing $\omega_{c}$ for the TD-NEFG method and the Landauer approach, respectively [80,147]. This effect can be tuned by the value of ΔT. However, independently of ΔT, the heat flux for both approaches become closer with increasing $\omega_{c}$.

#### 3.2.2. Atomistic System: Carbon-Based Molecular Junctions

**Figure 12:**
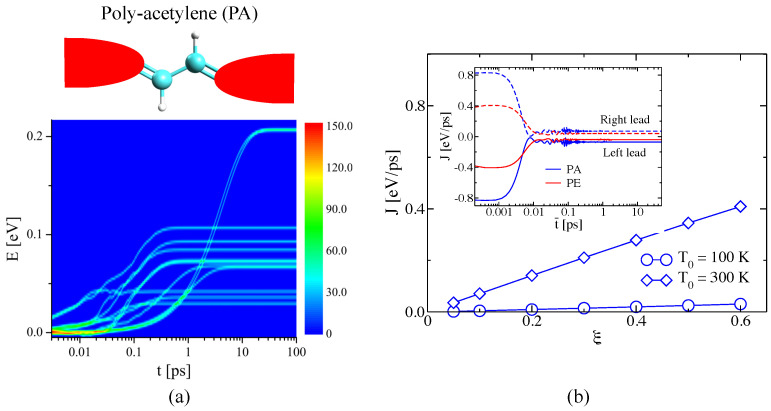
Time-dependent NEGF approach: (**a**) Energy density plot for molecular junctions made of poly-acetylene (PA) dimer. (**b**) Variation of the steady-state heat flux as a function of ξ for the PA dimer at different $T_{0}$. Inset: Dynamics of the heat flux for both leads for PA and PE dimers after applying a temperature bias of ΔT=60 K at $T_{0}=300$ K. The figure is reproduced with permission from Ref. [147]. Copyright 2018 American Chemical Society.

Medrano Sandonas et al. [147] showed that the TD-NEGF method reviewed in this section can be combined with atomistic methodologies for addressing the phonon dynamics in real systems. Due to the larger number of degrees of freedom, the matrix dimensions considerably increase and, hence, the computational cost. As typical examples, the work in Ref. [147] focused on poly-acetylene (PA, 4 atoms) and poly-ethylene (PE, 6 atoms) dimers connected to thermal baths (see Figure 12a for the case of PA dimer). The main difference between the two systems was the presence of double C bonds in PA compared with single bonds in PE. Both structural optimization and force constant calculations were performed with the Gaussian09 code [153]. $\Lambda_{\alpha }^{\left(0\right)}$ will thus take values corresponding to realistic bonds between the central region and the reservoirs (for more details, see [80]). For both junctions, a cut-off $\omega_{c}=100$ THz was used (roughly two times the maximum frequency of the vibrational spectrum). The number of poles in the auxiliary-mode expansion at 100 K, 300 K, and 500 K was 10, 8, and 4, correspondingly [80,147].

To gain a deeper understanding of the thermal properties in the transient state, the energy density D(E,t) was defined as:

(50) $$D\left(E,t\right)=\sum_{i=1}^{N}\left(1/\gamma \sqrt{2\pi }\right)\exp\left[-{\left(\left(E-E_{i}\right)/\gamma \sqrt{2}\right)}^{2}\right]$$

with γ=0.001 eV and $\left\{E_{i}\right\}$ the set of eigenvalues of Z(t). In Figure 12a, the results for PA dimer during thermal equilibration at $T_{0}=300$ K are displayed. As shown in the figure, all modes of the Z(t) matrix display very low energy at the beginning of the transient. The lowest lying modes gain then energy and reach a maximum at equilibrium. However, the eigenvalues in PE need a longer time to converge as compared to PA [147]. This difference arises from the different coupling strengths to the leads (related to the matrices $\Lambda_{\alpha }^{\left(0\right)}$). Consequently, the magnitude of the oscillations in the heat flux during the transient after applying a temperature bias is different, being larger for PA, as shown in the inset of Figure 12b. This difference in covalently bonded configurations also leads to a larger heat flux for the PA dimer [147]. Moreover, in agreement with linear response, the heat flux behaves nearly linear in ξ for different mean temperatures $T_{0}$ (see Figure 12b) [80,147].

## 4. Summary and Outlook

In the current review, we address selected applications of our recent implementations of quantum transport methodologies in low-dimensional materials. Hereby, we highlight the possibility to perform systematic investigations with atomic resolution, thus addressing material-specific problems for designing potential (nano)phononic devices.

We combined the NEGF formalism with the DFTB methodology to address quantum ballistic transport in various low-dimensional materials with atomistic resolution. This computational approach is implemented as a tool in the DFTB+ software. Although these systems may also be tractable using classical molecular dynamics, extensive parameterizations may be required to study different material combinations (here, machine learning approaches may be of interest). It is therefore more suitable to use the NEGF-DFTB approach, where the chemistry of the problem is naturally included in the first-principle calculation of the Hessian matrix. We showed that 2D puckered materials display strong thermal anisotropy due to their atomic structure, thus transporting heat preferably along the zigzag direction (higher phonon group velocity). As a next application, the influence of BN concentration and defect distribution on the thermal transport of BNC heteronanotubes was considered. Independently of the specific spatial BN distribution, the phonon transmission of pristine (6,6)-CNT is reduced at high frequencies after increasing the BN concentration. As a last application, we demonstrated that the vibrational features of molecular junctions can be exploited in conjunction with an appropriate choice of nanoscale thermal baths to implement a molecule-based phononic filter. This model offers the possibility of engineering different phonon filters based on the rich molecular chemical space. These three reviewed studies clearly demonstrate the potential of the PHONON tool to investigate nanoscale ballistic phonon transport.

In the last section, we present an atomistic method combining time-dependent NEGF with a first-principle based modeling to address phonon dynamics in nanoscale systems. The method is based on solving the equation of motion of the phonon density matrix with an efficient auxiliary-mode approach. The approach was applied to study thermal transport in the transient regime of a 1D chain, providing results in agreement with the Landauer formalism. By using density-functional theory to obtain the force constants and coupling matrices, the phonon dynamics of small molecular junctions was considered. Although the presented study is based on a Drude regularization of the spectral density, realistic scenarios can be easily addressed. This computational approach builds one of the first attempts to deal with time-dependent quantum phonon transport and it will allow studying various topical questions such as heat pumping, on a fully atomistic basis.

We are, however, not yet able to address physical effects such as thermal rectification from a fully quantum picture. Although rectification can be induced by structural asymmetries, phonon–phonon interactions play a dominant role, too. The latter are also crucial when dealing with phonon transport at high temperatures. An implementation combining NEGF with first-principles requires, besides computing the dynamical matrix as the basic input, third and fourth order anharmonic coefficients as well [76,78]. They contribute additional self-energies in the Green’s functions of the scattering region, and involve convolutions in frequency space of two-and three phonon Green’s functions. As a result, the problem needs to be solved self-consistently, thus considerably increasing the computational effort.

Another issue is the inclusion of electron–phonon coupling in the description of heat transport. Although it has already been implemented within the NEGF approach to address electronic transport [154,155,156,157,158], there are not many atomistic-based studies related to their impact on phonon transport. Since the interaction with the electronic system will provide an additional energy exchange channel, it will be of interest to elucidate how some of the effects discussed in this review as well as in other investigations, such as thermal rectification and phonon filtering, will be modified by the inclusion of electron–phonon interactions.

## Figures and Tables

**Table 1:** Calculated lattice constants of two-dimensional puckered materials along zigzag (ZZ) and armchair (AC) directions. For comparison, the lattice constants from other published theoretical studies are also given. In general, the DFTB lattice parameters agree quite well with those calculated by using DFT method, error ≤5%. The table is reproduced with permission from Ref. [117]. Copyright 2016 American Chemical Society.

Systems	Transport Direction		Other Works (ZZ, AC) [Å]
	ZZ [Å]	AC [Å]	
Phosphorene	3.49	4.34	(3.28, 4.43) [126] (3.32, 4.58) [127]
Arsenene	3.81	4.75	(3.68, 4.77) [128] (3.69, 4.77) [129]
SnS monolayer	3.93	4.51	(4.03, 4.26) [124] (4.01, 4.35) [130]

## Abbreviations

The following abbreviations are used in this manuscript:

CNT	Carbon nanotube
DFT	Density functional theory
DFTB	Density functional tight-binding
DOS	Density of states
EOM	Equation of motion
GF	Green’s functions
LDOS	Local density of states
NEGF	Non-equilibrium Green’s functions
NEMD	Non-equilibrium molecular dynamics
MD	Molecular dynamics
PA	Poly-acetylene
PE	Poly-ethylene
TD	Time-dependent
